# *In silico* genome wide identification and expression analysis of the WUSCHEL-related homeobox gene family in *Medicago sativa*

**DOI:** 10.5808/gi.22013

**Published:** 2022-06-30

**Authors:** Tianhui Yang, Ting Gao, Chuang Wang, Xiaochun Wang, Caijin Chen, Mei Tian, Weidi Yang

**Affiliations:** 1 Institute of Animal Science, Ningxia Academy of Agriculture and Forestry Sciences, Yinchuan 750002, China; 2 Branch Institute of Guyuan, Ningxia Academy of Agriculture and Forestry Sciences, Guyuan 756000, China; 3 Institute of Horticultural Science, Ningxia Academy of Agriculture and Forestry Sciences, Yinchuan 750002, China

**Keywords:** chromosome organization, *cis* element, *Medicago sativa*, synteny, WOX

## Abstract

Alfalfa (*Medicago sativa*) is an important food and feed crop which rich in mineral sources. The WUSCHEL-related homeobox (*WOX*) gene family plays important roles in plant development and identification of putative gene families, their structure, and potential functions is a primary step for not only understanding the genetic mechanisms behind various biological process but also for genetic improvement. A variety of computational tools, including MAFFT, HMMER, hidden Markov models, Pfam, SMART, MEGA, ProtTest, BLASTn, and BRAD, among others, were used. We identified 34 *MsWOX* genes based on a systematic analysis of the alfalfa plant genome spread in eight chromosomes. This is an expansion of the gene family which we attribute to observed chromosomal duplications. Sequence alignment analysis revealed 61 conserved proteins containing a homeodomain. Phylogenetic study sung reveal five evolutionary clades with 15 motif distributions. Gene structure analysis reveals various exon, intron, and untranslated structures which are consistent in genes from similar clades. Functional analysis prediction of promoter regions reveals various transcription binding sites containing key growth, development, and stress-responsive transcription factor families such as MYB, ERF, AP2, and NAC which are spread across the genes. Most of the genes are predicted to be in the nucleus. Also, there are duplication events in some genes which explain the expansion of the family. The present research provides a clue on the potential roles of *MsWOX* family genes that will be useful for further understanding their functional roles in alfalfa plants.

## Introduction

The WUSCHEL (WUS)-related homeobox (*WOX*) gene group is a subfamily that includes plant-specific transcription factors. WOX participates in multiple developmental activities, especially stem cell maintenance and organ development [1]. Typically, WOX amino acids fold into a DNA-binding domain termed the homeodomain, which is encoded by the homeobox (HB) DNA sequence and a homeodomain with 60–66 amino acid residues [2]. In higher plants, many homeodomains-containing transcriptional factor proteins have been identified in both monocots and dicots [3]. The HB protein superfamily is classified into six families. These classifications include homodomain-leucine zipper (HD-Zip); plant homeodomain (PHD)-finger; BELL; zinc finger-homeodomain (ZF-HD); WOX; and KNOTTED1-like-homeobox (KNOX) [4].

Of these homeodomains, members of the WUS-related gene family have been comprehensively identified or predicted in many plants, such as Arabidopsis, maize, soybean, rice, etc. [5-7]. The model eudicot plant Arabidopsis (*Arabidopsis thaliana*) contains 15 WOX proteins, which are classified into three clades based on evolutionary relationships i.e., a modern/WUS clade, an intermediate clade, and an ancient clade [8].

Most WUS members are involved in multiple developmental processes, including embryonic development, embryonic polarization, meristematic stem cell maintenance, lateral organ development, seed formation, and regeneration of isolated tissues and organs [9]. Aside from plant growth and development, another important factor to consider is plant response to environmental stresses. In order to survive extreme environmental stress, plants have evolved multiple mechanisms as a defense strategy against external signals by modulating gene expression [10]. Transcription factors such as WRKY, MADS-BOX, NACs, BHLH, and HSF, among many others, have been confirmed to play key roles in regulating plant response to abiotic stresses and are listed in the plant stress transcription factor database (http://caps.ncbs.res.in/stifdb). Recent evidence suggests that *WOX* genes also play a role in the regulation of abiotic stress resistance. A poplar WOX11/12a gene, for example, has been shown to play an important role in drought tolerance [11]. Overexpression of WOX13 under the rab21 promoter increased drought stress tolerance in rice [12]. HOS9 has been shown in Arabidopsis to regulate cold stress tolerance [13]. Even though several members of the WOX family have been cloned and functionally studied, little is still known about these members and their roles in many plants.

Alfalfa is a popular food and feed crop that is farmed all over the world. It is typically collected as hay, but it can also be processed into silage, grazed, or supplied fresh. The development and quality of alfalfa is limited due to numerous difficult conditions such as a lack of water, cold temperatures, and excessive salt, and productivity is lowered by at least 10%–20% [14]. The entire genome data of the autotetraploid cultivar XinJiangDaYe were released in 2020, resulting in a chromosome-level genome assembly with 32 genes [15]. This gathering will give a wealth of information for identifying important stress-related genes and genetically engineering alfalfa stress tolerance.

In this study therefore, we analyzed the *WOX* gene family in alfalfa based on available genome sequence. We identified 34 genes of the WOX gene family based on a genome-wide scan approach and predicted their functions by combining the analysis of the phylogenetic tree with that *cis*-promoting element. We also studied the chromosomal location and gene structures as well as subcellular localization. This study provides further insight into the structure and function of the *WOX* gene family in alfalfa and is useful for their further genetic studies.

## Methods

### Identification of WOX members in alfalfa

The latest versions of the genome annotations of alfalfa were retrieved from the genome assembly (https://www.alfalfatoolbox.org/). Previously reported *Arabidopsis* WOX full-length and homeodomain amino acid sequences were retrieved from The Arabidopsis Information Resource (TAIR, http://www.arabidopsis.org/) aligned with MAFFT v5.3 [16] and then subjected to HMMER v3.0 [17] for building HMM (hidden Markov models) profiles. The HMM profiles were applied to perform HMM search against the annotated alfalfa protein databases with an E-value cutoff of 1e-5. Furthermore, using both the full-length and homeodomain amino acid sequences of *Arabidopsis* WOXs, a BLASTP search with an E-value cutoff of 0.01 was carried out to identify additional potential WOX proteins. The protein sequences from the two methods outlined above were merged, and redundant entries were manually eliminated. Pfam (https://pfam.xfam.org/) and SMART (http://smart.embl.de/) [18] were used to check the hit sequences for the presence of the homeobox domain.

### Sequence alignment and phylogenetic analysis

MAFFT v5.3 was used to align the full-length and homeodomain amino acid sequences of *Arabidopsis* WOXs and probable WOX members from the alfalfa species using the default parameters with manual editing. Based on the alignment data, two distinct approaches to constructing phylogenetic trees were used. First, using MEGA software 6.06 [19] under the following parameters: Poisson correction, pairwise deletion, and bootstrap values, a neighbor-joining tree was created from the alignment of full-length amino acid sequences of *Arabidopsis* and alfalfa WOX members (1,000 replicates). Second, the alignment of homeodomain amino acid sequences of *AtWOXs* and alfalfa WOXs was used to create a Bayesian inference (BI) tree [20]. With the help of ProtTest 2.4, the same JTT + G model was chosen. MrBayes (http://mrbayes.sourceforge.net/) [21] was used to analyze the BI tree for 2,500,000 generations, with trees sampled every 1,000 generations and a burn-in of 625 while a FigTree v1.4.0 was used to view these tree files.

### Gene chemical structure analyses

Using both coding and genomic sequences, exon-intron structures were analyzed and depicted using the Gene Structure Display Server (GSDS, http://gsds.cbi.pku.edu.cn/). MEME 4.9.1 (http://meme-suite.org/) was used to identify conserved motifs in *Arabidopsis* WOXs and alfalfa WOX proteins, and WebLogo (http://weblogo.berkeley.edu/logo.cgi) [22] was used to visualize them. The following parameters were set: the distribution of motif occurrences was set to zero or one per sequence; the maximum number of motifs was set to eight; the optimal motif width was set to six and 100; and the optimal number of sites for each motif was set to two and 200.

### Chromosome location and gene duplication analysis

The distribution of members of the alfalfa WOX family on the alfalfa chromosomes was examined using the *WOX* gene annotation information in the alfalfa genome database. The duplicate gene pairs were searched from the plant genome duplication database server (http://chibba.agtec.uga.edu/duplication/index/locket). The amino acid sequence of the partly repeated *MsWOX* gene was determined using the Clustalw program [23].

### Subcellular localization prediction

A web-based interface for predicting the subcellular localization was retrieved from pre-built ngLOC model database (http://ngloc.unmc.edu/). To generate predictions, WOX protein sequences were supplied in the FASTA format and alfalfa species set as default. the MLCS (Multi-Localization Confidence Score) [24] was searched which reflects if the top two locations are predicted within a close probability margin.

### Synteny and Ka/Ks analysis

BLASTn was also used to see if Darwinian positive selection impacted the evolution of the *MsWOX* genes in alfalfa and its diploid progenitors. MCScanX was used to look for synteny blocks containing *WOX* genes between various alfalfa and *Arabidopsis* genomes and/or subgenomes. Each *WOX* gene was successfully mapped onto the relevant *Arabidopsis* chromosomes, according to information collected from gff3 files of genome annotation data. To illustrate the blocks and collinearity of homologous gene pairs, the CIRCOS software was utilized. The nonsynonymous to synonymous substitution ratios (Ka/Ks) of all orthologous, paralogous, and homoeologous gene pairs were estimated using the program KaKs Calculator version 2.0 using the model average and model averaging methods based on coding sequence alignment.

### Tissue-specific expression analysis

RNA-sequencing (RNA-seq) data from Phytozome 12 were used to examine the expression patterns of *MsWOX* genes in distinct tissues. The study covered six different tissues: root, nodule, leaves, flower, pre-elongated stem, and elongated stem. We used the FPKM (fragments per kilobase of transcript per million mapped fragments) data to create a heatmap.

## Results

### Identification and phylogenetic analysis of *MsWOX* genes in alfalfa

Homeodomain sequences from previously identified *Arabidopsis* WOX proteins were used to construct an HMM profile, which was then used as a query to perform HMM searches across related protein databases to identify *WOX* family genes in alfalfa. BLASTP searches were also carried out to identify other potential WOX proteins, with full-length and homeodomain sequences of known *Arabidopsis* WOX proteins serving as queries. To eliminate duplicate sequences, manual reconstruction was employed. The use of Pfam and SMART analyses to confirm the presence of the homeodomain in each candidate protein increased the dependability of these candidate sequences. Consequently, we discovered 34 candidate *WOX* genes, which corresponded to previous study (Table 1). These genes encoded proteins ranging in size from 14,609.5 to 153,183.84 kDa, with an instability index of 32 to 66.91, an aliphatic index of 49.78 to 88.07, and isoelectric point (pI) values ranging from 5.33 to 9.79 (Table 1).

### Sequence alignment and phylogenetic analysis

**Fig. 1.**
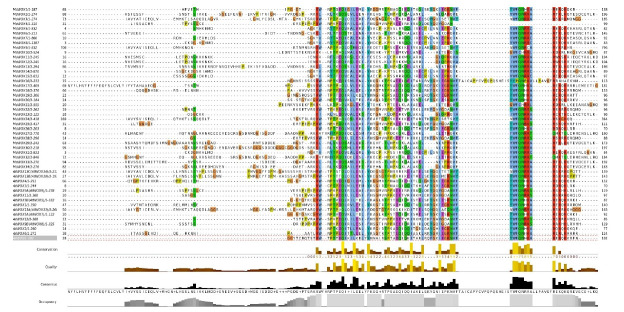
Characteristics of the WUSCHEL-related homeobox (WOX) domain sequences in alfalfa plants. The logo of the conserved WOX domains was analyzed using the WebLogo program.

WOX proteins are plant-specific proteins with a conserved homeodomain. As a result, the sequences of these proteins were aligned to produce sequence logos in order to determine if the domain is conserved in *MsWOX* members. The findings of the alignment indicated that the homeodomain was highly conserved among the genes (Fig. 1). The homeodomain featured a helix-loop-helix-turn-helix structure and was 61 amino acids long. The homeodomain sequences revealed three highly conserved residues in particular: Q in helix 1, L in helix 2, and W in helix 3. R and E in helix 1, I and P in helix 2, and Q and F in helix 3 are among the other highly conserved amino acid residues in the homeodomain. Surprisingly, all the above-mentioned amino acid residues were found to be highly conserved across all the homeodomain sequences analyzed.

**Fig. 2.**
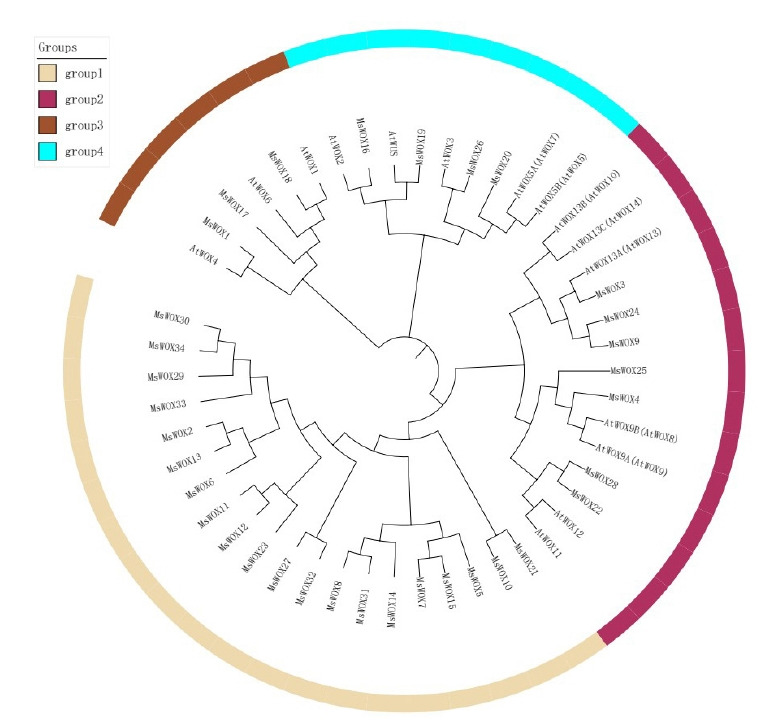
Phylogenetic tree showing the relatedness of alfalfa WUSCHEL-related homeobox (WOX) proteins to that of Arabidopsis.

In order to examine the evolutionary connection of alfalfa and *Arabidopsis* WOX members, the MEGA v5.0 platform was used to create a phylogenetic tree using their full-length sequences. The members of the alfalfa clade were given new names based on the original *Arabidopsis* names discovered in the same group (Fig. 2). The WOX members may be divided into four groups: group 1, group 2, group 3, and group 4. Group 1 has the most *Arabidopsis* and alfalfa species. While group 4 was the smallest clade in each analyzed species, it had nine WOX members, indicating that WOX members from *Arabidopsis* and alfalfa species may have an evolutionary link.

### Gene motif analysis

**Fig. 3.**
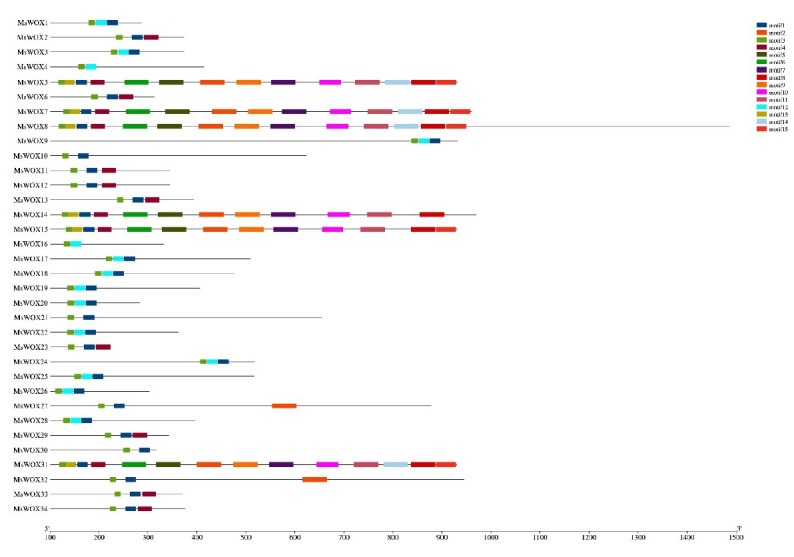
Distributions of conserved motifs. Motifs were mined using the MEME software and depicted as 15 different color boxes.

The conserved motif analysis validated the differences between the *MsWOX* genes in the ancient lineage and other clades. A total of 15 motifs were discovered and labeled as motif 1 through motif 15. Motif 3 was found in every *MsWOX* protein and was shown to encode the WOX homeodomain. Furthermore, genes from the same evolutionary group have nearly identical gene architectures. For example, motif 4 was shared by all members of group 1. Furthermore, all *WOX* genes in group 2 had just three motifs, whereas *WOX5, WOX6, WOX7, WOX8*, and *WOX31*, which all belong to the first group, had the most motifs. The location of these conserved motifs revealed more about the *WOX* genes in alfalfa plants. Except for *MsWOX9*, all *MsWOX* members in group 1 have a WUS-box; this suggests that *MsWOX* members from various clades may be engaged in distinct biological processes in alfalfa (Fig. 3).

### *MsWOX* exon-intron organization

**Fig. 4.**
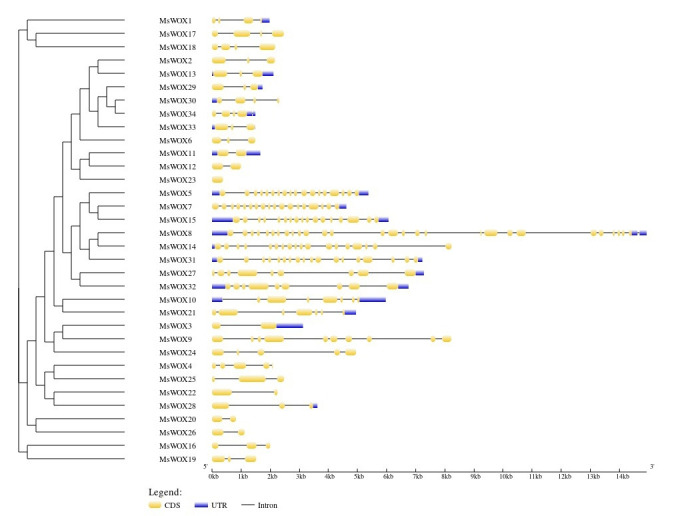
Phylogenetic tree showing grouping and the structural classification of *MsWOX* genes in *Medicago sativa*.

The exon-intron structure is closely related to the function of the corresponding gene, and it reflects the evolutionary relationship of multigene families when combined with phylogenetic analysis. To gain a better understanding of the *MsWOX* gene structure in alfalfa, the exon/intron organization was studied. According to the findings, all *MsWOX* genes have zero to five exons. Surprisingly, both *MsWOX13a/13b* have three exons in the ancient clade. Generally, the number of exons in *MsWOX* genes varies between clades. *MsWOX*8 of group1, for example, had the most exon number of 29, whereas *MsWOX23* of group 2 had only one exon. Small differences in gene characteristic are also observed within members of the same group. For example, *MsWOX23* had no intron, whereas *MsWOX22, MsWOX26, MsWOX20, MsWOX12,* and *MsWOX1* all had one despite belonging to the same clade. Untranslated regions were present in 17 members, the vast majority of whom belonged to group 1. But generally, except for MsWOX8 which featured complicated exon-intron structures and significant variation in the number of introns; and *MsWOX23* which lacked introns and untranslated regions, exon/intron architectures were found to be similar among WOX members within the same subgroup (Fig. 4). This organization of exons and introns in a gene family could give information about its evolution.

### Chromosome organization

**Fig. 5.**
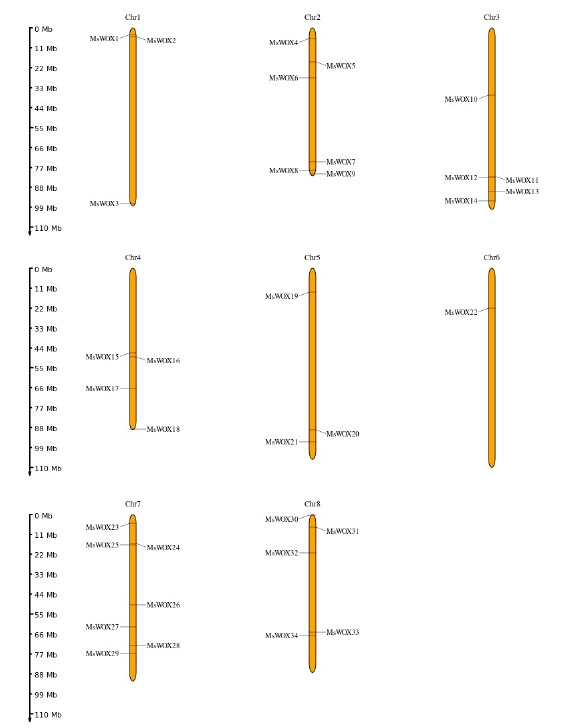
Chromosomal locations of *MsWOX* genes in alfalfa. The number of chromosomes was labeled on the top of each chromosome. The location of each WUSCHEL-related homeobox (*WOX*) genes was marked on the chromosome. The left pane is the chromosome size.

All the *MsWOX* genes were mapped into eight chromosomes. The number of genes per chromosome ranged from one in chromosome 6 to seven in chromosome 7. Gene members from similar group were located on same chromosomes. For example, chromosome 1 with three genes, *MsWOX1, MsWOX2* of were all located on chromosome 1. *MsWOX3* however is from group whose orthologous members such as *MsWOX23, MsWOX24, MsWOX25, MsWOX26, MsWOX27, MsWOX28*, and *MsWOX29* are all located on chromosome 7. There are duplications in *MsWOX9* (*MsWOX9A* and *MsWOX9B*) and *MsWOX5* (*MsWOX5A* and *MsWOX5B*) as well as a triplication *MsWOX13* (*MsWOX13A, MsWOX13B,* and *MsWOX13C*). Chromosome 6 had only one gene *MsWOX22* (Fig. 5).

### Collinearity and Ka/Ks analysis

**Fig. 6.**
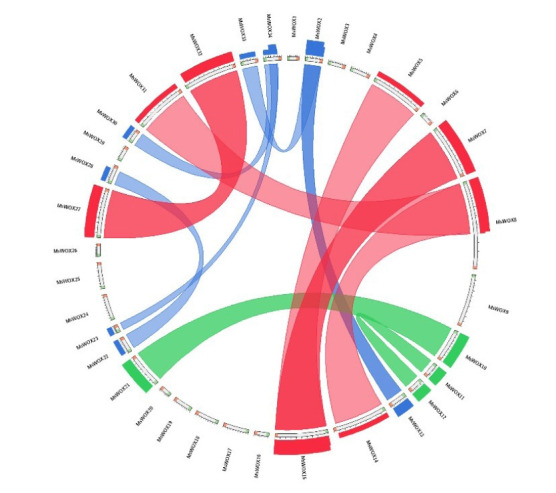
Chart depicting the collinear relationships of *MsWOX* genes in alfalfa. The inner-colored lines show syntenic blocks in homoeologous chromosomes among alfalfa WUSCHEL-related homeobox (*WOX*) genes.

To illustrate the locus linkage of homologous *MsWOX* genes among genomes, synteny analysis of WOX genes in alfalfa and its diploid progenitors was undertaken. As shown in Fig. 6, syntenic genes were a couple of genes connected by a line, whereas those connected by lines of the same color signified the same type of *MsWOX* gene, such as *MsWOX10* and *MsWOX21*. As a result, we can see that many chromosomes in all four genomes/subgenomes were connected by the same-colored line, indicating that these genomes/subgenomes were evolutionarily related and that the *WOX* genes were so crucial that most of them survived polyploidization.

The nonsynonymous (Ka), synonymous (Ks), and Ka/Ks ratios were calculated to assess selection pressure among duplicated *WOX* gene pairs. Ka/Ks = 1 implies that genes evolved in a neutral fashion; Ka/Ks > 1 or Ka/Ks 0 imply that genes were being positively selected or purified, respectively. All duplicated *WOX* gene pairs in alfalfa and its diploid progenitors exhibited Ka/Ks values less than one (Supplementary Table 1), with *MsWOX15-MsWOX5* having the lowest value of 0.0475257 and *MsWOX13-MsWOX15* having the highest value of 0.818763 (Supplementary Table 1).

### *Cis*-regulatory element and transcription factor binding

**Fig. 7.**
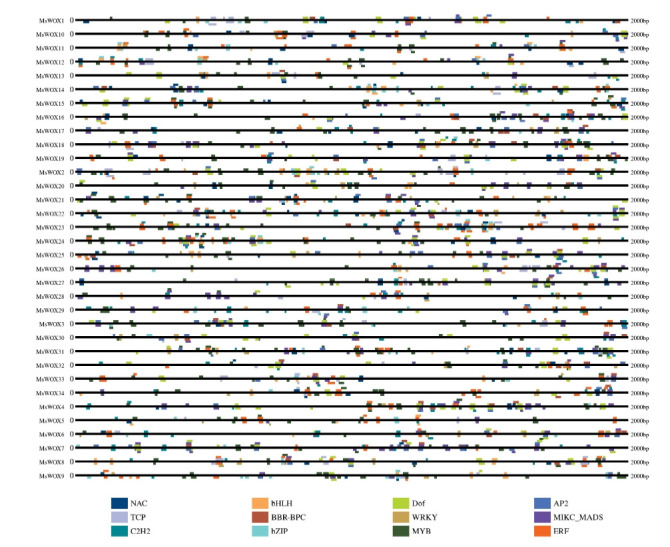
A diagram depicting transcription binding sites and the various transcription factors of cis-elements in *Medicago* sativa plant.

*Cis*-regulatory elements are unique DNA sequences found upstream of gene coding sequences that govern the expression of stress-responsive genes by interacting with transcription factors. Thus, the *cis*-elements in the putative promoter regions of the 34 *MsWOX* genes were investigated to further study the possible characteristics of the *MsWOX* family genes that are engaged in plant regulatory network management. Consequently, we discovered that MYB included the most *cis*-regulatory elements, followed by Dof, ERF, Bzip, MIKC-MADs, and AP2 (Fig. 6, Supplementary Table 2). Furthermore, the *cis*-elements were found in all of the genes. The ERFs were found on *MsWOX23* and *MsWOX10*, whereas the MYBs were found on *MsWOX16*. TCP is the least represented element, with only one member in *MsWOX11* (Fig. 7, Supplementary Table 2).

### Tissue-specific expression analysis

**Fig. 8.**
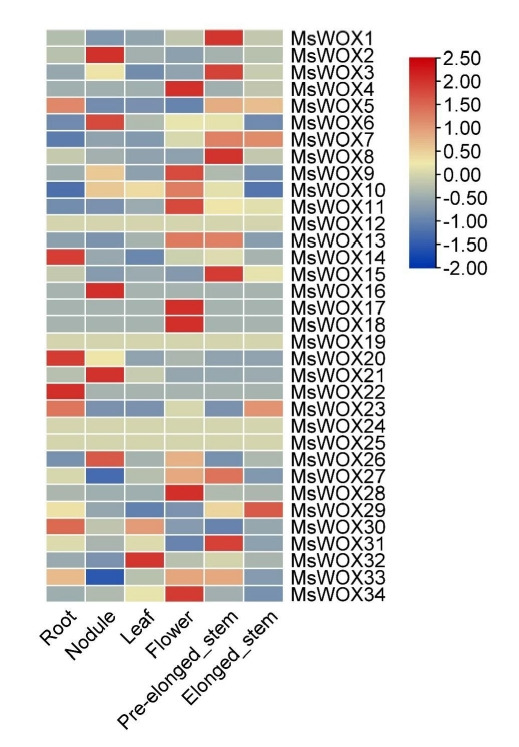
*MsWOX* gene expression patterns in different tissues. According to Genome Database *RNA-seq* data, the heatmap depicts gene expression patterns of *MsWOX* genes in 11 distinct organs. The color scale represents the levels of gene expression. The blue represents a low expression level, whereas the red color represents a high expression level.

The heatmap revealed that *MsWOX12, MsWOX19, MsWOX24*, and *MsWOX25* had nearly identical expression patterns in all tissues. Their expressions varied between -0.5 and 0.5. Some gene expressions were found to be tissue-specific. Flowers, for example, had the highest representation of highly expressed genes among the organs, while the leaf, with just *MsWOX32* strongly expressed, had the lowest number of genes. In each tissue, just a few genes were expressed. *MsWOX1, MsWOX3, MsWOX8, MsWOX7, MsWOX8, MsWOX13, MsWOX14*, and *MsWOX31* were all found in significant concentrations in the pre-elongated stem. The leaf, on the other hand, has only one highly expressed gene which is *MsWOX32*. These tissue expression patterns particular to subfamilies may be linked to gene activities. Heat maps highlighted the expression patterns of the paralogous pairings, and we discovered that most of the paralogous pairs with high sequence similarity had comparable expression patterns (Fig. 8).

**Fig. 9.**
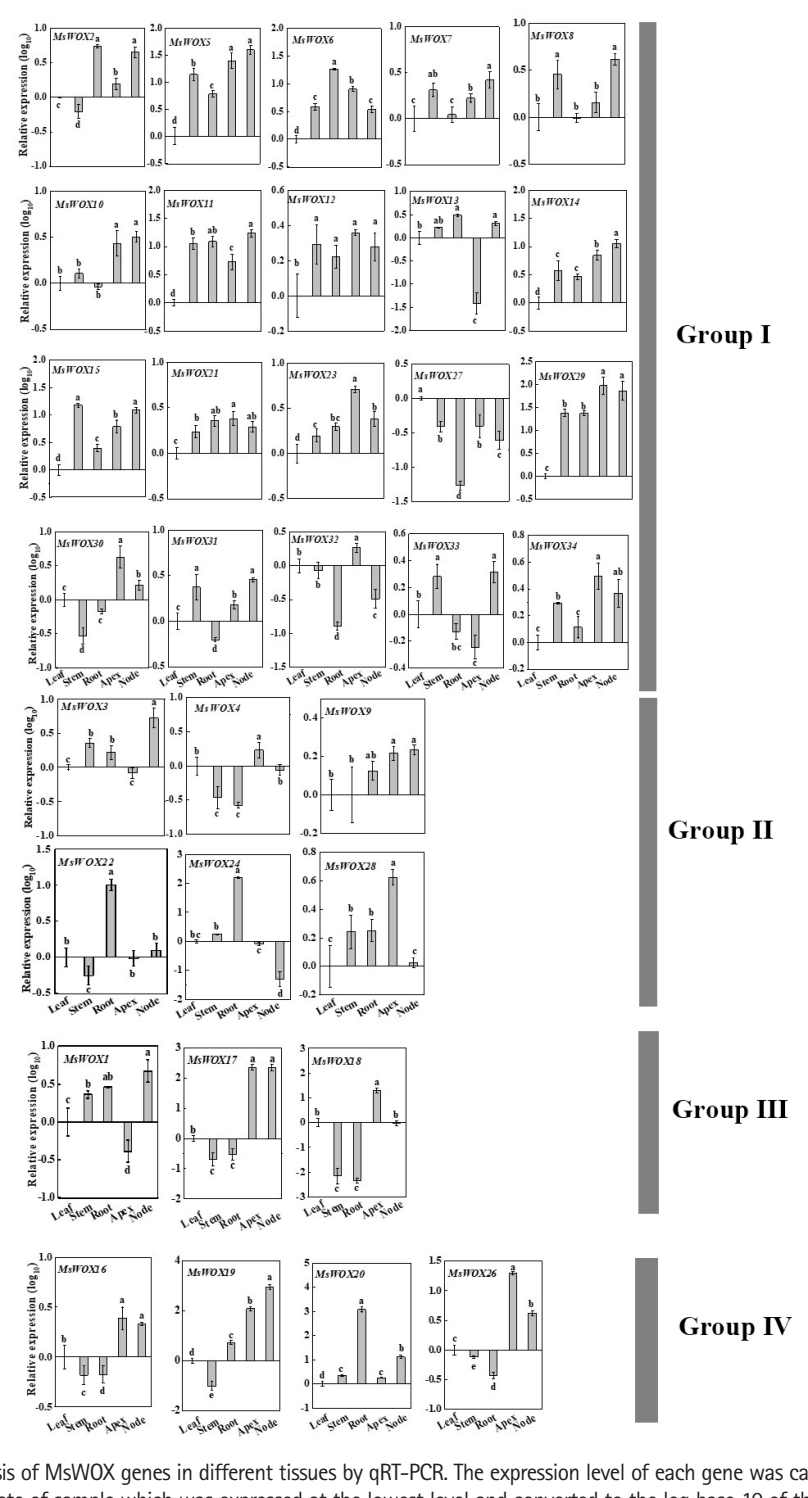
Expression analysis of MsWOX genes in different tissues by qRT-PCR. The expression level of each gene was calculated relatively to the average biological replicate of sample which was expressed at the lowest level and converted to the log base 10 of the value. Different letters indicate statistically significant differences when analyzed by One-way ANOVA and a multiple comparison using Tukey’s test at p ≤ 0.05.

To verify and enrich the expression profiles of *MsWOX* genes, real-time reverse transcription-PCR (qRT-PCR) analysis of 28 selected genes was conducted in five different tissues. The gene expression pattern detected by qRT-PCR was generally consistent with the RNA-seq data. *MsWOX20* and *MsWOX22* expression, for example, was both increased in the roots. Furthermore, these genes were intimately involved in a variety of organ-specific developmental processes (Fig. 9).

## Discussion

As sequencing technology improves, so does the efficiency and accuracy of sequencing. As a result, more and more species’ genomic data are being published. This provides data that may be used to investigate gene structure and function prediction in the context of whole-genome comparison and identification. Here, putatively, 34 *MsWOX* genes from alfalfa species were identified and analyzed in this study. Phylogenetic analysis using two different methods revealed similar topologies, which were endorsed further by exon-intron organization analysis, motif assessment, and functional prediction. The alfalfa WOX proteins, along with *Arabidopsis* WOX proteins, were categorized into four well-organized groups. Genes from the same clade could have similar roles. For example, WUS-box in WOX11 and WOX12 are both involved in root development under abiotic stress in Brassica [25].

This study's findings, combined with those from previous ones, suggest that the *WOX* genes in the *Medicago* species are highly conserved in structure and function in plant development and stress resistance. Unique features in some WOX members are also visible, which is due in part to the extensive expansion of some subgroups via gene duplication, as well as gene loss in some other subgroups following species divergence. A conserved homologous domain of 61 amino acid residues was discovered in all 34 WOX transcription factor family members in alfalfa, according to protein structure analysis. When compared to other species’ WOX proteins, we see a “helix-loop-helix-turn-helix” homeodomain structure. This domain is essential for DNA recognition and binding [26]. The homeodomain’s most conserved amino acids are the final amino acid at the second helix, the last amino acid residue of the turn structure, and the amino acid residue V of the middle position of the second helix structure [27]. According to our observation, the three amino acid residues are in the interior of the homeodomain, indicating that they may play an important transcription role as previously suggested [28].

Moreover, the angle of formation of “helix-turn-helix” structure in ancient branches is smaller than that of intermediate clades and modern clades, which may lead to functional changes in the evolution process of WOX transcription factor. The residues may correlate and play a role in the evolutionary process [3]. In addition, the WUX-box with LRP domain might play important role in the adventitious shoot organogenesis [29]. We also observe a conserved motif in the C-terminal region through multiple sequence alignment; other studies, accordingly, have found that the IC-WOX domain may be involved in root evolution [30].

As the binding sites of transcription factors, *cis*-acting elements in the promoter of the gene determine its expression patterns [31]. In our study, a series of pant development-and stress-related cis-acting elements were detected in the promoter of *MsWOXs*. The maximum number of cis-acting elements were MYBs and ERF elements, which play important role in plant growth, development, and response [32,33].

Whole-genome duplication (WGD) has been proven in several studies to impact the number of gene families in the genome [34]. WGD events in the alfalfa plant genome may have influenced the amount of MsWOX members. The old lineage of *WOX* genes, for example, is represented by group 1. Through multiplication, we see expansion events in WOX9 (*WOX9A* and *WOX9B*) and WOX5 (*WOX5A* and *WOX5B*). In group 2, duplicated *Arabidopsis* WOX13 members were found together with WOX9 duplicated members, whereas the other WOX5 was found on a separate branch of the phylogenetic tree. The fast evolution of *WOX* genes is suggested by these gene expansions. Gene duplication may have happened after the alfalfa species was separated from other plants, resulting in the isolated members [15]. The protein motifs and exon-intron organization of the isolated group of *WOX*9 members were also discovered to be distinct. It was revealed that the separated members had bigger gene sizes and longer introns than the original members. Furthermore, all isolated *WOX*13 subgroup members from alfalfa species lacked motif 3, which was present in all other *WOX*13 subgroup members. Surprisingly, isolated *WOX*9 binds more MYBs and ERF transcription factors, according to a cis-element analysis. This is consistent with the discovery that the MYB and ERF families have developed swiftly and selectively in response to diverse environmental pressures [35-37].

In addition, both *MsWOX9* and *MsWOX13* members have a highly preserved motif layout. When compared to ancient and contemporary clade subgroups, the gene architectures of these two subgroups have the largest variation, with exon counts ranging from 3‒5 in the WOX9 subgroup genes and 2–4 in the *MsWOX11* subgroup genes. Previously, it was discovered that genes in the enlarged *MsWOX5* play an important role in drought tolerance in the Jatropha plant [38]. Different functions of *MsWOX9* proteins from other species have also been discovered, confirming this theory. Lie et al. [39] found that *Arabidopsis* WOX9/STIP in combination with WOX8/STPL is necessary for embryo patterning and vegetative SAM maintenance, whereas Petunia EVERGREEN/WOX9 is required for inflorescence growth and architecture [40]. All of this demonstrated that the intermediate clade was rapidly evolving, with significant changes in gene architecture, expression patterns, and likely gene functions among its members, which may be influenced by environmental factors like desiccation.

Except for *MsWOX23*, all WOX members of the current lineage have a homeodomain and a WUS box. The gene lacks the second exon which harbors the WUS box. The WUS box has been identified as the AtWUS functional domain essential for SAM cell identity induction and maintenance [41]. Also, the motif organization and gene architectures, as well as the functional prediction of current clade members within the same subgroup, were found to be identical, except for *MsWOX23*, which has no homologue in alfalfa species. Therefore, despite being evolutionary like *MsWOX11* and *MsWOX12*, its function may be different due to the absence of the essential WUS box.

In conclusion, we identified 34 *WOX* genes in alfalfa using bioinformatics methods and a genome-wide database. According to structural features, the *MsWOX* genes are classified into four groups (group 1, group 2, group 3, and group 4) with 20 *MsWOX* genes belonging to group 1, seven in group 2, four in group 3, and three in group 4. According to chromosomal mapping, all the *MsWOX* genes were distributed on eight chromosomes. Collinearity analysis of *MsWOX* genes indicated considerable collinearity in 81.3% of alfalfa *WOX* genes. Numerous *WOX* genes are implicated in multiple gene duplication events, according to the complicated linear connection. Also, some *WOX* genes in alfalfa had no introns, while most *MsWOX* genes in the same subgroup exhibited comparable patterns of exon length, intron number, and conserved motifs, according to structural analysis. Cis elements and transcription factor binding analysis of the *MsWOX* gene revealed high abundance of MYBs, ERFs, and AP2 transcription factors whose number varied widely per motif. TCP was among the least. These findings indicate that *MsWOX* genes may have a role in development under abiotic stress.

## Figures and Tables

**Table 1.** Characteristics of alfalfa *WOX* gene family

Gene name	Gene location	A.A No.	Weight (Da)	Isoelectric points	Instability index	Aliphatic index	GRAVY
*MsWOX1*	Chr1:3806424‒3808395	187	21561.3	9.79	57.8	61.98	‒0.858
*MsWOX2*	Chr3:85829292‒85830284	274	30,925.1	8.52	51.31	70.11	‒0.795
*MsWOX3*	Chr7:4947090‒4947467	274	31,285.2	6.43	49.72	70.77	‒0.678
*MsWOX4*	Chr7:79679330‒79681060	315	34,498.8	6.98	48.96	69.9	‒0.43
*MsWOX5*	Chr8:241473‒243772	832	91,168.9	5.92	51.79	86.07	‒0.151
*MsWOX6*	Chr8:7362896‒7370122	213	24,537.7	6.67	48.71	82.86	‒0.888
*MsWOX7*	Chr8:22027384‒22034137	860	93,547.5	5.99	45.86	86.83	‒0.075
*MsWOX8*	Chr8:67803988‒67805460	1387	153,184	6.11	44.9	88.07	‒0.21
*MsWOX9*	Chr8:69416480‒69417965	832	94,413.6	5.02	52.81	77.34	‒0.742
*MsWOX10*	Chr1:5066220‒5068376	524	59,633	5.99	52.97	57.67	‒1.055
*MsWOX11*	Chr1:100681352‒100684467	245	29,008.2	5.33	66.91	52.53	‒1.284
*MsWOX12*	Chr2:6284516‒6286598	245	29,020.2	5.33	65.36	53.71	‒1.284
*MsWOX13*	Chr2:19355940‒19361319	294	33,344	6.57	56.22	63.37	‒0.914
*MsWOX14*	Chr2:28821465‒28822950	870	95,414.2	6.86	44.34	86.25	‒0.166
*MsWOX15*	Chr2:76808477‒76813101	832	91,266.1	5.99	46.75	87.12	‒0.11
*MsWOX16*	Chr2:81776107‒81791035	233	25,965	5.58	48.85	68.58	‒0.545
*MsWOX17*	Chr2:83669682‒83677900	409	47,097.2	8.52	51.12	67.04	‒0.589
*MsWOX18*	Chr3:38501335‒38507307	376	43,056.9	8.42	56.52	62.34	‒0.838
*MsWOX19*	Chr3:85344929‒85346585	306	34,532.9	6.41	60.02	57.42	‒0.888
*MsWOX20*	Chr3:94036221‒94038325	184	21,209.7	6.53	56.58	49.78	‒0.79
*MsWOX21*	Chr3:99347579‒99355806	555	62,699.3	6.31	56.33	56.43	‒1.064
*MsWOX22*	Chr4:48397488‒48403556	262	27,602.4	8.81	50.49	59.24	‒0.476
*MsWOX23*	Chr4:50875345‒50877341	125	14,609.5	6.97	32	71.76	‒1.077
*MsWOX24*	Chr4:69003312‒69005774	418	47,489.3	9.37	48.54	83.06	‒0.609
*MsWOX25*	Chr4:92442615‒92444780	417	46,428.5	5.53	50.26	62.59	‒0.621
*MsWOX26*	Chr5:13693575‒13695090	203	23,472.4	9.23	64.49	60.54	‒0.736
*MsWOX27*	Chr5:93003502‒93004323	778	85,772.5	6.17	50.97	81.22	‒0.321
*MsWOX28*	Chr5:99882444‒99887399	296	32,286.5	7.67	52.38	72.74	‒0.317
*MsWOX29*	Chr6:23108175‒23110420	243	27,513.9	6.75	47.85	65.47	‒0.659
*MsWOX30*	Chr7:16766076‒16771035	218	24,141.7	6.53	50.3	59.95	‒0.944
*MsWOX31*	Chr7:17564121‒17566590	831	91,815.2	6.15	46.72	87.21	‒0.152
*MsWOX32*	Chr7:51962820‒51963935	846	93,017.4	5.84	49.06	82.48	‒0.29
*MsWOX33*	Chr7:64567276‒64574554	270	30,175.7	6.96	53.5	62.52	‒0.824
*MsWOX34*	Chr7:75163974‒75167582	276	31,344.4	7.67	65.18	66.78	‒0.752

WOX, WUSCHEL-related homeobox; A.A, amino acid; GRAVY, grand average of hydropathicity index.

## Supplementary Materials

Supplementary Table 1. Ka and Ks values of MsWOX gene family members.

Supplementary Table 2. TF binding predict of MsWOX gene family memebers.
